# Speech and Voice Response to a Levodopa Challenge in Late-Stage Parkinson’s Disease

**DOI:** 10.3389/fneur.2017.00432

**Published:** 2017-08-22

**Authors:** Margherita Fabbri, Isabel Guimarães, Rita Cardoso, Miguel Coelho, Leonor Correia Guedes, Mario M. Rosa, Catarina Godinho, Daisy Abreu, Nilza Gonçalves, Angelo Antonini, Joaquim J. Ferreira

**Affiliations:** ^1^Faculty of Medicine, Instituto de Medicina Molecular, University of Lisbon, Lisbon, Portugal; ^2^Department of Speech Therapy, Escola Superior de Saúde de Alcoitão, Estoril, Portugal; ^3^Campus Neurológico Sénior, Torres Vedras, Portugal; ^4^Department of Neurosciences, Hospital Santa Maria, Centro Hospitalar Lisboa Norte, Lisbon, Portugal; ^5^Laboratory of Clinical Pharmacology and Therapeutics, Faculty of Medicine, University of Lisbon, Lisbon, Portugal; ^6^Center for Interdisciplinary Research Egas Moniz (CiiEM), Instituto Superior de Ciências da Saúde Egas Moniz, Almada, Portugal; ^7^Parkinson Disease and Movement Disorders Unit, IRCCS San Camillo Hospital Foundation, Venice-Lido, Italy; ^8^Department of Neurosciences, University of Padua, Padua, Italy

**Keywords:** Parkinson’s disease, late stage, levodopa, speech, voice

## Abstract

**Background:**

Parkinson’s disease (PD) patients are affected by hypokinetic dysarthria, characterized by hypophonia and dysprosody, which worsens with disease progression. Levodopa’s (l-dopa) effect on quality of speech is inconclusive; no data are currently available for late-stage PD (LSPD).

**Objective:**

To assess the modifications of speech and voice in LSPD following an acute l-dopa challenge.

**Method:**

LSPD patients [Schwab and England score <50/Hoehn and Yahr stage >3 (MED ON)] performed several vocal tasks before and after an acute l-dopa challenge. The following was assessed: respiratory support for speech, voice quality, stability and variability, speech rate, and motor performance (MDS-UPDRS-III). All voice samples were recorded and analyzed by a speech and language therapist blinded to patients’ therapeutic condition using *Praat* 5.1 software.

**Results:**

24/27 (14 men) LSPD patients succeeded in performing voice tasks. Median age and disease duration of patients were 79 [IQR: 71.5–81.7] and 14.5 [IQR: 11–15.7] years, respectively. In MED OFF, respiratory breath support and pitch break time of LSPD patients were worse than the normative values of non-parkinsonian. A correlation was found between disease duration and voice quality (*R* = 0.51; *p* = 0.013) and speech rate (*R* = −0.55; *p* = 0.008). l-Dopa significantly improved MDS-UPDRS-III score (20%), with no effect on speech as assessed by clinical rating scales and automated analysis.

**Conclusion:**

Speech is severely affected in LSPD. Although l-dopa had some effect on motor performance, including axial signs, speech and voice did not improve. The applicability and efficacy of non-pharmacological treatment for speech impairment should be considered for speech disorder management in PD.

## Introduction

Parkinson’s disease (PD) patients are classically affected by hypokinetic dysarthria, characterized by hypophonia and dysprosody, that worsens with disease progression due to breathing, phonation, and articulation dysfunction (1–3). Speech disorders affect nearly 90% of PD patients and have a negative impact on functional communication, which in turn contributes to decreased quality of life (4, 5). Symptoms vary from a soft and breathy voice that lacks modulation in volume (monoloudness) and fundamental frequency (monopitch or monotone) resulting in flat speech melody (dysprosody), with pitch breaks, lack of rhythm and pace of speech, number of pauses, reduced stress, and imprecision in consonant articulation, to a voice that is neither audible nor intelligible (6–9).

The effect of levodopa (l-dopa) on the quality of speech is inconclusive given that it is also influenced by each patient’s speech profile. Some studies report on a slight improvement of intonation, vowel articulation, and speech intelligibility (10–13), while others show no significant effect (14, 15) as measured during an acute l-dopa challenge. Nevertheless, speech is generally considered to be an “l-dopa-resistant” axial motor symptom of PD (16). Axial impairment is preponderant among PD patients in the latest disease stage (17), although no data are currently available on the effect of l-dopa on speech among late-stage PD (LSPD) patients. The purpose of this study was to assess the clinical and active modifications of speech and voice after an acute l-dopa challenge in an LSPD population.

## Patients and Methods

### Design and Recruitment

We performed a cross-sectional study in a consecutive sample of LSPD patients recruited during 12 months from the movement disorders outpatient clinic of a tertiary university hospital (Hospital Santa Maria, Lisbon, Portugal). PD was defined according to the UK Brain Bank criteria (18), whereas LSPD was defined as PD patients with either a Schwab and England score <50 (MED ON) or a Hoehn and Yahr stage (HY) >3 (MED ON) (19). The Local Ethics Committee approved the study. All subjects gave written informed consent in accordance with the Declaration of Helsinki.

### Assessment of Patients

Late-stage PD patients were first assessed in the practically defined “MED OFF” condition and then 60–90 min after l-dopa intake in the best “MED ON” condition. For the l-dopa challenge, each patient took her/his usual morning l-dopa equivalent dose plus 50% (supramaximal dose = 150%). l-Dopa equivalent daily dose was calculated according to recognized standard conversions (20). Details of the l-dopa challenge have been previously reported (19).

The following parameters were assessed during both MED OFF and MED ON: (a) motor performance by means of the MDS-UPDRS part III (21); (b) severity of dyskinesias using the Modified Abnormal Involuntary Movement Scale (mAIMS); (c) respiratory support for speech (time duration of vowel/a/prolongation); (d) voice quality [fundamental frequency (*F*_0_)]; (e) voice stability (pitch break time and jitter); (f) voice variability [SD of speaking *F*_0_ during sentences (Sentence *F*_0_SD)]; and (g) speech rate (syllables/s). Each participant had to perform several vocal tasks that consisted of the following: (i) sustained phonation of the vowel/a/at a comfortable pitch and loudness and (ii) repeating an 8-word, 14-syllable standard statement/declarative sentence, “A Maria comprou-me um mapa do papel branco” [translation: Mary bought me a map of white paper]; and (iii) reading 5 words and 5 sentences. Tasks were selected from the European Portuguese version of the Frenchay Dysarthria Assessment version 2 (22). However, due to the low level of cooperation of LSPD patients, we adopted an 8-word (14 syllables) declarative sentence (syntactically simple) that in European Portuguese is expected to have a low level of voice variability compared to complex sentences or text reading, which are normally used for this task.

Patients were seated and instructed by a neurologist to sustain the vowel/a/at a comfortable pitch and loudness as long as they could. A demonstration was made by the clinician before the patient performed each vocal task. There were no time limits for each participant and he/she was asked to repeat the task if the examiner was not fully satisfied with patient’s performance.

All voice samples were recorded in a room in a home environment using a tabletop unidirectional microphone (Fame, MS-1800S) attached to a preamplifier (M-Audio Fast Track Pro, preamp, USB) and a desktop computer running *Audacity software version 2.1.2* (Free software Foundation Europe, Hamburg, Germany).

Two separate perceptual files were completed using *Audacity software* version 2.1.2 with all the stimuli presented at the same sound pressure levels and with a 500 ms silence between single words and sentences.

MDS-UPDRS parts II and IV were used to assess the impact of motor symptoms on activities of daily life and l-dopa-induced motor complications, respectively. PD with dementia was diagnosed according MDS Task Force recommendations (23).

### Data Analysis

All voice samples were copied to a computer (down sampled to 24 kHz, 16 bits, mono), edited into individual files and screened for extraneous noise using *Audacity* by a speech language therapist with expertise in experimental phonetics and who was not involved in data gathering and was blind to the participants’ demographics and clinical status.

Acoustically, the waveform, spectrogram, pitch, intensity, and the formants of each sustained vowel were visually observed using the *Praat* 5.1 software (24) downloaded from http://www.praat.org.

The vowel/a/mean and SD *F*_0_ (Hz), jitter (local, %) and harmonic-to-noise-ratio (dB) were analyzed with a moving window with at least 1-s using voice report in the *Praat* software.

The following parameters were analyzed: (a) *Respiratory support for speech*. Duration (s) was measured as the total period between the onset and offset of each sustained vowel/a/and the breath(s) during speech in the sentence “A Maria comprou-me um mapa de papel branco”; (b) *Voice (pitch) quality*. The average *F*_0_ (Hz) was analyzed in all vowels in the two moments. Vowel/a/was perceptually analyzed by a speech language therapist for pitch and loudness level along the production (mainly high or low); (c) *Voice (pitch) stability*. The assigned acoustic parameters were as follows: Pitch breaks (no pitch contour) time (seconds) and jitter (local, %). Vowel/a/was perceptually analyzed by considering the pitch and loudness stability (maintained, increased, decreased or uncontrolled); (d) *Voice variability*. Variability was considered as speech *F*_0_SD in hertz in the sentence (Sentence *F*_0_SD). At baseline (MED OFF) the *F*_0_SD (Hz) was also analyzed; and (e) *Speech rate*. Speech rate of the sentence “A Maria comprou-me um mapa de papel branco” [Mary bought me a map of white paper], total number of orthographic syllables divided by total time duration (including pauses).

### Statistical Analysis

Descriptive statistics of demographic, clinical, and therapeutic data were provided for continuous (median and interquartile range [IQR, 25th–75th percentile]) and categorical (count and percentage) variables.

Voice and speech characteristics at baseline (MED OFF) of LSPD patients, considering men and women separately, were compared to the available normal values of healthy age-matched subjects, although no statistical analyses were performed.

The acute effect of l-dopa on voice and speech was calculated by comparing the median duration of the vowel/a/, average *F*_0_, pitch breaks duration, jitter, S*F*_0_SD, and speech rate between MED OFF versus MED ON conditions. Comparisons were made using the Wilcoxon’s signed-rank test.

Spearman’s rank correlation coefficient was used to assess the association between: (a) respiratory support for speech, voice quality, voice stability, voice variability, speech and disease duration, and motor impairment (MDS-UPDRS-III)/axial motor impairment (sum of items 3.1, 3.10–3.12 of the MDS-UPDRS-III); (b) speech rate and freezing (item 3.11 of the MDS-UPDRS-III).

Two group comparisons (women versus men) were performed using the Mann–Whitney *U*-test.

#### Reliability of Analyses

To evaluate test–retest reliability of acoustic measurements the sustained vowel/a/for an average *F*_0_ was run twice. A satisfying test–retest reliability was found (*R* = 0.722, *p* < 0.001, Pearson test), only one single-speech-task cycle was performed for the definite acoustic measurements.

A *p* value < 0.05 was considered significant. The analysis of the results was carried out by means of SPSS 21.0 (SPSS, Chicago, IL, USA).

## Results

### Clinical Data

Twenty-seven LSPD patients were recruited for speech and voice analyses. Three were excluded due to their inability to perform the required tasks (one anarthric patient and two due to severe dementia). Demographic and clinical data of the 24 LSPD patients are detailed in Table 1.

**Table 1:** Values are presented as median [IQR, 25th–75th percentile] if no otherwise specified.

Patients data	LSPD (*n* = 24)	LSPD Male (*n* = 14)	LSPD Female (*n* = 10)	*p*-Value
Age (years)	79 [71.5–81.7]	77.5 [70.7–81.2]	79 [73.5–85]	ns
Age at disease onset (years)	64.5 [54.5–69.5]	62.5 [55–67]	65 [51.5–71.5]	ns
Disease duration	14.5 [11–15.7]	13.5 [8.7–17]	15 [11.7–17.2]	ns
Education (years)	4 [4–11]	4 [4–12]	5 [4–10.5]	ns
S&E (ON/OFF)	40/35 [40–40.7/22.5–40]	40/30 [40–40/40–40]	40/30 [27–50/17.5–50]	ns
HY (ON/OFF)	4 [2–4]/4 [2–4.75]	3 [2–4]/3 [2–4]	4 [4–5]/4 [4–5]	ns
PDD [*n* (%)]	14 (58%)	10 (71%)	4 (40%)	ns
MMSE	22.5 [21.2–25]	22.5 [22–24.2]	22.5 [16–27.2]	ns
MMSE (demented/non-demented)	22 [17–23.7]/25 [23–26.7]	22 [21.7–24.2]/23 [22.2–25.2]	17 [13–19.5]/27 [25–28.5]	
LEDD (mg)	1,037 [902–1,272]	1,100 [990–1,303]	905 [742–1,257]	ns
MDS-UPDRS-II	31 [27–38]	32 [29.2–38.5]	30 [20.5–38]	ns
MDS-UPDRS-III (MED ON/MED OFF)	50 [40–54]/64 [52–77]	50 [42.5–55.2]/61 [53–76]	50 [37.5–62.5]/64 [48–79.5]	ns
Axial sign (MED ON/MED OFF)	8 [6–13]/10 [7–13]	8 [6–13]/10 [7–13.2]	8 [6.5–12]/10 [7–13.5]	ns
MDS-UPDRS-IV	4 [2–9.5]	5 [2–8.5]	4 [0–11.2]	ns

*LEDD, l-dopa equivalent daily dose; PDD, Parkinson’s disease with dementia; MMSE, mini mental state examination; S&E, Schwab and England score; HY, Hoehn and Yahr stage; ns, non-significant; LSPD, late-stage PD*.

*p-Value is the results for male versus female scores’ comparison*.

There were no differences in demographic or clinical variables between men and women (Table 1). Indeed, they presented similar MDS-UPDRS II–III–IV scores, axial signs score, SE and HY stages, although women had a slightly, but not statistically significant, worse HY stage, and more men were demented although not statistically significant (Table 1).

### Baseline (MED OFF) Voice and Speech Characteristics

No differences were found between men and women for breath support and voice stability at baseline (MED OFF) (Table 2). Voice quality differed between men and women at baseline, although this difference has been noticed in vocally healthy subjects (gender effect) and the values were also similar to vocally healthy subjects (25) (Table 2). Values of respiratory breath support (26) and pitch break time (24) of LSPD patients appeared worse when compared to the normal values of healthy age-matched subjects, stratified for gender (Table 2). Mean jitter values were in the normal range (Table 2), although results were borderline for men and SD showed a tendency for higher values (27). In contrast, *F*_0_SD (28) was in the normal range (Table 2). However, this result was partially expected as we use a very syntactically simple sentence.

**Table 2:** Values for late-stage PD patients are presented as median [IQR, 25th–75th percentile].

	Parkinson’s disease patients (*N* = 24)		Normal value	
**Respiratory support for speech**				
Vowel duration (s)	5.8 [4.4–11.5.8]		22.97 (1.1)^b^	
**Voice stability**				
Pitch break time (s)	1.24 [0.2–2.6.1]		NA^a^	
Jitter (%)	0.8 [0.5–1.1]		≤0.5–1%	
**Voice variability**				
Sentence *F*_0_SD (Hz)	2.4 [1.6–4]		2–4 Hz	

**Voice quality (Hz)**	**Male (*N* = 14)**	**Female (*N* = 10)**	**Male**	**Female**

*F*_0_	125 [104–152]	202 [160–226.8]	128 (36)^c^	198 (44)^c^

*Values for healthy subjects are presented as mean (SD), as reported in literature (25–28)*.

*F_0_, fundamental frequency; Sentence F_0_SD, SD of speaking F_0_ during sentences*.

*^a^Not available (healthy voices should have no trouble in maintaining voicing during a sustained vowel. Thus is 0% of voice breaks. No standard values are available)*.

*^b^Normal value for vowel duration is referred to a healthy population aged between 71 and 80 years old*.

*^c^Normal value for voice quality is referred to a healthy population aged between 55 and 80 years old*.

A positive moderate correlation was found between disease duration and voice quality (*R* = 0.51; *p* = 0.013) and a negative one with speech rate (*R* = −0.55; *p* = 0.008). Motor impairment (MDS-UPDRS-III) had a moderate significant correlation with respiratory support for speech (*R* = −0.43; *p* = 0.045) and pitch break time (*R* = −0.565; *p* = 0.006). No correlations were found between voice and speech features and axial motor impairment, neither between speech rate and freezing. When analyzing by gender (men and women separately) such correlations were partially maintained: (a) voice quality and disease duration: men (*R* = 0.5; *p* = 0.079) and women (*R* = 0.36; *p* = 0.2); (b) speech rate and disease duration: men (*R* = −0.7; *p* = 0.003) and women (*R* = −0.2; *p* = 0.5); (c) respiratory support for speech and MDS-UPDRS-III: men (*R* = 0.64; *p* = 0.017) and women (*R* = −0.7; *p* = 0.029).

### l-Dopa Acute Challenge Test

No differences between men and women were found when comparing motor, voice, and speech variables during both MED OFF and MED ON, except for voice quality (*F*_0_), as was expected (see Table 2 for voice characteristics of healthy subjects). Thus, further analyses were carried out by taking into consideration the whole LSPD sample and not stratifying by gender.

#### Motor Response

The median l-dopa dose for the test was 375 mg [IQR: 277–375]. The median MDS-UPDRS-III score was 64 [IQR: 52–77] in MED OFF and 50 [IQR: 40–54] in MED ON, with a significant median improvement of 20% [IQR: 11.5–32%] (*p* < 0.001) (Table 3). Sub-analysis of MDS-UPDRS-III scores for axial signs showed a significant median improvement after l-dopa intake for all the subitems, except speech (Table 3). 3 patients (12.5%) had mild dystonic dyskinesias in MED OFF, while 12 (50%) presented slight-moderate choreic dyskinesias in MED ON.

**Table 3:** Values are presented as median [IQR, 25th–75th percentile].

LSPD patients (*N* = 24)			
	MED OFF	MED ON	*p*-Value
**MDS-UPDRS-III**	64 [52–77]	50 [40–54]	**<0.001**
Speech	2 [1–3]	2 [1–3]	0.83
Freezing of gait	3 [1–4]	2 [0–3]	**<0.05 (0.01)**
Postural stability	3 [2–4]	3 [2–3]	**<0.05 (0.014)**
Gait	3 [2–4]	3 [2–3]	**<0.05 (0.01)**
Axial signs	10 [7–13]	8 [6–13]	**<0.05 (0.01)**
**HY**	4 [2–4.75]	4 [2–4]	0.7
**mAIMS**	0	1 [0–6.75]	**0.04**
**Voice respiratory support for speech**			
Vowel duration (s)	5.8 [4.4–11.5]	7 [3.6–10.6]	0.6
**Voice stability**			
Pitch break time	1.2 [0.2–2.6]	0.8 [0.07–2.5]	0.9
Jitter	0.8 [0.5–1.1]	0.7 [0.4–1]	0.5
**Voice quality**			
*F*_0_	154 [123–209]	162 [147–203]	0.2
**Voice variability**			
Sentence *F*_0_SD	31 [19–51]	29 [20–40]	0.5
Speech rate	5 [3.6–5.6]	5 [4.2–5.7]	0.2

*Statistical significant results are in bold. Axial signs: sum of item 3.1, 3.10–3.12 of the MDS-UPDRS-III. p-Value is the results of MED OFF versus MED ON scores*.

*mAIMS, Modified Abnormal Involuntary Movement Scale; F_0_, fundamental frequency; Sentence F_0_SD, SD of speaking F_0_ during sentences; LSPD, late-stage PD; HY, Hoehn and Yahr stage*.

#### Voice and Speech Response

None of voice and speech variables changed significantly after l-dopa intake (Table 3).

Equally, separate analysis of non-demented and demented patients showed no modification of speech and voice variables following l-dopa intake.

## Discussion

The purpose of this study was to explore the l-dopa response of speech in the late stage of PD. In order to do this a population of LSPD patients underwent an l-dopa challenge while performing specific vocal tasks during both MED OFF and MED ON conditions. No effect of l-dopa was found on speech and voice by means of both automated analysis and clinical evaluation, although patients had a moderate positive motor response, even present for some axial signs, with the exception of speech. Such a discrepancy in l-dopa responsiveness between speech and other axial signs has been reported only in one previous speech study in advanced PD patients (14) and suggests that speech together with balance and postural problems could be listed among l-dopa resistant axial sign appearing with disease progression.

Despite not performing a case-controlled study, by comparing MED OFF speech and voice characteristics of our patients with normative values of the general population we found a severe impairment of respiratory support for speech and voice stability, as already reported elsewhere (6, 12). We chose to make this comparison in the MED OFF condition because it more accurately reflects the parkinsonian state of patients. Rigidity associated with PD can often lead to disruption of respiratory processes which serve to generate air pressure for speech (10). Respiratory support for speech may be measured through vowel prolongation, and a decrease by an average of fifty percent in vowel prolongation has been reported for PD patients when compared to normal healthy speakers (10). Among our LSPD patients, vowel prolongation was more affected, even in the absence of dyskinesias that can affect respiratory control (11). Equally, voice stability, i.e., ability to maintain a consistent voice during a stable/sustained vowel with laryngeal muscle effort, is impaired in MED OFF, as shown by an increase in pitch break time and the tendency for jitter increment. Moreover, a tendency for worsening voice quality and speech rate was highlighted with disease duration. Voice quality and voice variability values in MED OFF were in the normal range although the most plausible cause for this finding is methodological, which might have resulted in falsely normal values for voice quality and variability: we have chosen a declarative sentence for voice variability analysis that is syntactically too simple to capture this feature; equally, we assessed voice quality using mean *F*_0_ instead of *F*_0_SD which is usually more appropriate but not possible to analyze in our patients due to the technical quality of the recordings. Interestingly, no correlations were found between speech rate and freezing. These data are apparently in contrast with the recent findings of Ricciardi and colleagues that showed lower scores in the articulation, intelligibility, rate/prosody section of the Dysarthria Profile in PD patients with freezing of gait (FOG), as assessed by the New FOG questionnaire, if compared to PD patients without FOG (29). However, in our study, different methodological measures have been adopted in order to assess both speech rate and FOG. Moreover, Ricciardi and colleagues included younger PD patients, belonging to several HY stages, thus a more heterogeneous PD sample, scarcely comparable to our LSPD patients.

Our sample of LSPD patients still presented moderately good motor response to l-dopa (20% of the MDS-UPDRS-III) when compared to our previous report (19), and the frequency of dementia was slightly lower (52%) (19). The exclusion of patients who could not speak at all or who could not properly understand the tasks would have surely created bias. Thus, our sample may represent a subset of LSPD patients who present a slightly better clinical state compared to other reports (30, 31). Nevertheless, even if an influence of dyskinesias on speech performance cannot be excluded (11), speech showed no improvement after l-dopa intake, whether it was measured clinically or with automated analysis that explored the respiratory support for speech (vowel duration), voice stability, variability and quality, and speech rate. De Letter et al. evaluated respiratory features among 25 non-demented PD patients during an l-dopa challenge and reported a slight improvement of sustained vowel phonation (11). However, due to the clinical differences with our sample, i.e., older patients with longer disease duration and worse l-dopa response, these results may not be comparable with those published by De Letter et al. Concerning voice stability and variability, if we assume that hypokinesia of the voice apparatus is the major pathological mechanism of monopitch speech in PD (32, 33), *F*_0_SD should improve after l-dopa intake and should decline further during the disease course. However, data on voice stability/variability improvement after l-dopa are inconsistent, and previous reports have also failed to show a response of *F*_0_SD or jitter to dopaminergic therapy (12, 15, 34). This finding may be related to the usual worse response of axial muscles to l-dopa.

A lack of improvement in speech quality (*F*_0_) and speech rate after l-dopa or apomorphine has already been described in earlier PD stages (12, 14, 15, 35). We report similar data in LSPD patients, although we have to consider that our patients did not present with a severe impairment of voice quality in MED OFF. Thus, an improvement would not be expected. A slight improvement of speech rate after l-dopa intake has been found in only nine PD patients with optimal l-dopa responsiveness and a non-severe impairment of speech at baseline, as assessed by the UPDRS-III (34). However, Ho et al. concomitantly reported on a decay of rate improvement during the speech testing tasks (34). Thus, it is likely that improvement in speech rate is not maintained during the tasks.

Several factors can contribute to the lack of speech and voice responsiveness to l-dopa in PD patients, especially in the late disease stage.

Speech production is essentially a series of skilled motor gestures that require upstream central coordination mediated by cerebral networks for speech production. Indeed, the globus pallidus (GP) produces a phasic burst of activity that triggers the supplementary motor area neural discharge, allowing cortical motor set for movement preparation and subsequent execution (34). In PD, the impairment of GP activity alters those mechanisms, resulting in diminished movement amplitude and impairment of movement sequencing. Such a process affects speech production as well as body movement, and a correlation between speech hypophonia/speech intensity and severity of body bradykinesia has been suggested (34). l-Dopa has been shown to have an effect on preparatory motor set, resulting in hypokinesia improvement, but failed to affect movement sequencing (36). Likewise, concerning speech, while still controversial, a few studies have reported on a slight l-dopa positive effect on loudness (speech intensity), intonation (speech variability), and speech rate (12, 34) at least in early-advanced PD stages. Conversely, speech stability and variability seem to be definitively impervious to dopaminergic therapy (9, 12). Interestingly, and contrary to previous suggestions, we did not find neither an improvement of speech intensity or rate with l-dopa nor a correlation between speech and voice severity and motor symptoms that still respond to l-dopa, namely, bradykinesia and rigidity. These findings may support a non-dopaminergic involvement in speech neurocircuitry as already supposed in earlier disease stages (35), and this is even more likely in LSPD (37). Alternatively, a higher dose of l-dopa could be needed to improve speech, as is often the case with gait dysfunction. The usual absence of significant rigidity in late-stage patients (19, 31) may also have contributed to the lack of correlation between speech intensity and motor impairment. Furthermore, we have to consider that a loss of striatal responsiveness is related to disease progression and is likely responsible for a decrease or loss of clinical response to dopaminergic therapy of several motor symptoms (19), which also probably affects speech responsiveness. Finally, motor speech production also depends on the appropriate function of peripheral nervous system (7). Dysfunction of speech articulation may also be partly attributed to muscular denervation and atrophy, resulting in respiratory muscles impairment whose function does not improve with l-dopa as recently shown in a sample of PD patients in HY 2–4 (38). Such muscle impairment is presumably even more severe among older PD patients who have a worse motor status as our sample.

Our findings highlight the need for alternative non-dopaminergic/non-pharmacologic treatments to improve communication of LSPD patients. For instance, the Lee Silverman Voice Treatment has shown some efficacy in the treatment of voice and speech problems of PD patients (7). However, its applicability to LSPD patients should be verified due to the level of collaboration that it requires and the degree of disability of those patients.

### Study Limitations

Some limitations of our study must be highlighted. Due to the clinical disability of LSPD patients, recordings were performed in a home environment and not in a laboratory setting. This implied accepting samples varying in context, over different time periods, and recorded in non-standard environments. Nevertheless, the quality and reliability of the recordings were evaluated by a speech language therapist. Patients’ disabilities can also have influenced choice of tasks. For instance, we selected a simple task for voice variability assessment, which was probably not sensitive enough to detect l-dopa effect in voice/intonation variability, or voice variability defect at baseline. Finally, clinical assessment of patients was not blinded. However, there was concordance between clinical and automated assessments of speech.

### Conclusion

To the best of our knowledge, this is the first report on l-dopa response of speech and voice in a sample of LSPD patients by means of both a clinical rating scale and automated analysis. Speech is severely affected among LSPD patients, as already reported for PD patients in earlier disease stages (1, 4).

Although l-dopa still had some effect on motor performance, including some axial signs, we found no improvement in speech and voice. Clinical management and research should consider the applicability of non-pharmacological treatments for speech and voice impairment among LSPD patients.

## Ethics Statement

The Local Ethics Committee of the “Centro Hospitalar Lisboa Norte, Lisbon, Portugal,” approved the study. All subjects gave written informed consent in accordance with the Declaration of Helsinki.

## Author Contributions

MF and IG: substantial contributions to the conception and design of the work, drafting the work; final approval of the version to be published and agreement to be accountable for all aspects of the work in ensuring that questions related to the accuracy or integrity of any part of the work are appropriately investigated and resolved; RC and MC: substantial contributions to the conception and design of the work; final approval of the version to be published and agreement to be accountable for all aspects of the work in ensuring that questions related to the accuracy or integrity of any part of the work are appropriately investigated and resolved; LG and MR: substantial contributions to the interpretation of data for the work; revising the work critically for important intellectual content; and final approval of the version to be published and agreement to be accountable for all aspects of the work in ensuring that questions related to the accuracy or integrity of any part of the work are appropriately investigated and resolved; CG: substantial contributions to the conception or design of the work, revising it critically for important intellectual content; final approval of the version to be published and agreement to be accountable for all aspects of the work in ensuring that questions related to the accuracy or integrity of any part of the work are appropriately investigated and resolved; DA and NG: substantial contribution to the analysis of data, revising the work critically for intellectual content, final approval of the version to be published, and agreement to be accountable for all aspects of the work in ensuring that questions related to the accuracy or integrity of any part of the work are appropriately investigated and resolved; AA and JF: substantial contributions to the interpretation of data for the work, revising the work critically for important intellectual content, final approval of the version to be published, and agreement to be accountable for all aspects of the work in ensuring that questions related to the accuracy or integrity of any part of the work are appropriately investigated and resolved.

## Conflict of Interest Statement

The authors declare that the research was conducted in the absence of any commercial or financial relationships that could be construed as a potential conflict of interest.
